# Measuring the impact of dental service quality on revisit intention using an extended SERVQUAL model

**DOI:** 10.3389/froh.2024.1362659

**Published:** 2024-04-12

**Authors:** Rayan Sharka, Lamer Sedayo, Majd Morad, Jameel Abuljadayel

**Affiliations:** ^1^ Oral and Maxillofacial Surgery Department, Faculty of Dental Medicine, Umm Al-Qura University, Makkah, Saudi Arabia; ^2^ Dental Teaching Hospital, Faculty of Dental Medicine, Umm Al-Qura University, Makkah, Saudi Arabia; ^3^ Preventive Dentistry Department, Faculty of Dental Medicine, Umm Al-Qura University, Makkah, Saudi Arabia

**Keywords:** service quality, patient perceptions, revisit intention, dental care, dental services

## Abstract

**Background:**

The pursuit of quality services can lead to both service enhancement and increased motivation to visit dental centers for oral health treatment.

**Aims:**

The purpose of this study is to investigate the effect of dental center service quality factors on revisit intention among adult patients by applying an extended service quality model (SERVQUAL).

**Methods:**

This cross-sectional study was conducted between September and November 2023 in the outpatient waiting areas and clinical settings of Umm Al-Qura University Dental Teaching Hospital (UQU-DTH). A sample of 355 patients was invited by the convenience sampling method. The data was collected through a validated Arabic version of the extended SERVQUAL questionnaire. A hierarchical regression analysis was used to assess the incremental effects of the extended SERVQUAL factors on the intention of patients to revisit the UQU-DTH while controlling for demographic variables. Cronbach alpha was used to examine the internal consistency of each model factor.

**Results:**

A total of 330 completed responses were received, with a 93% response rate. The findings indicated that demographic variables such as age and level of education contribute to some extent but become negligible when the extended SERVQUAL factors are included in the model. Moreover, the extended SERVQUAL model factors substantially improved the model. Three factors were found to positively and significantly affect the revisit intention, namely, “staff-related factors,” “cost-effectiveness,” and “responsiveness.” Overall, the model explained 65.6% of the variance in the revisit intention (*R*^2^ = 0.656, *p* < 0.001).

**Conclusion:**

The findings present a unique model that may be used to better understand the factors that influence patients' intentions to revisit dental centers in an educational setting. Additionally, it identified elements that dental center quality management needs to prioritize and address.

## 1 Introduction

The assessment of the quality of dental healthcare has become a key concern in the provision of care, and it is acknowledged that patient feedback and perceptions are vital elements of these assessments, as they help to find gaps and offer ongoing improvements to the service provided and its outcome (1, 2). The dental healthcare sector is transitioning towards a consumer-centric model, where dentists are seen as service providers and patients are viewed as consumers (3). Quality health-care service entails delivering prompt, successful, and reliable services based on the most up-to-date clinical recommendations and guidelines (4). Moreover, it aims to satisfy patients by offering distinctive service elements like availability, accessibility, affordability, competence, and timeliness, as assessed by the SERVQUAL model (5). Given the current emphasis on clinical governance and patient engagement in providing excellent oral healthcare, it is essential to address quality concerns raised by patients in a suitable manner (6).

Dental clinics at dentistry schools often aim to achieve a harmonious equilibrium between fulfilling patient requirements and providing practical instruction to students. Understanding patients' revisit intentions in such educational settings is crucial, as it will impact and ensure their usage of services. Evidence demonstrates that patients who expressed higher levels of satisfaction with dental services and treatment had improved adherence, reduced the frequency of missed visits, and experienced decreased levels of worry (7–10). Hence, it is important to get data on patients' perceptions in order to accurately assess the quality of the service provided and then set appropriate improvement plans accordingly.

The healthcare system in Saudi Arabia is undergoing substantial changes and leadership reorganization to achieve considerable improvements in quality, efficiency, and safety. These initiatives seek to revolutionize the healthcare industry by guaranteeing long-term funding, sufficient availability, and continuous improvement in the quality of services offered to patients (11). The Umm Al-Qura University Dental Teaching Hospital (UQU-DTH) is a modern dental facility that serves as both a teaching and clinical center for dentistry in Makkah City in the western region of Saudi Arabia. The center was founded with the purpose of training undergraduate students as well as postgraduate students. The center's objective is to cultivate a cohort of dentists who possess a high degree of expertise and proficiency while also delivering dental treatment across several dental disciplines. Nevertheless, Service quality has rarely been comprehensively evaluated by patients.

Scholars have created and examined many service quality models (12). A frequently used approach is SERVQUAL, which assesses service quality using five factors (5). Another approach, known as SERVPERF, likewise assesses service quality using five factors but places more emphasis on the performance of health organizations (13). Furthermore, there exists a model with two predictors that analyzes the impact of both expectation and performance on customer satisfaction (12). Additionally, it should be emphasized that service quality models may need adaptation or creation tailored to distinct businesses and settings. These numerous models provide frameworks for evaluating and enhancing service quality in numerous contexts. Some studies were conducted to ascertain the factors that impact revisit intention for health services, including the correlation between SERVQUAL model factors and revisit intention (14–16). However, none of these studies were conducted in Saudi Arabia. Moreover, previous studies carried out in Saudi Arabia utilizing the SERVQUAL model were conducted in a medical context (17–20); there were few studies focused on the quality of dental care services. Also, dental teaching hospitals have not been extensively studied with respect to the service quality provided. Therefore, this study aims to investigate the effect of hospital service quality factors on revisit intention among adult patients by applying an extended SERVQUAL model. The following research question guides the methodology: Which service quality factors have a salient influence on patients intentions to revisit UQU-DTH?

## 2 Conceptual development and hypotheses

**Figure 1:**
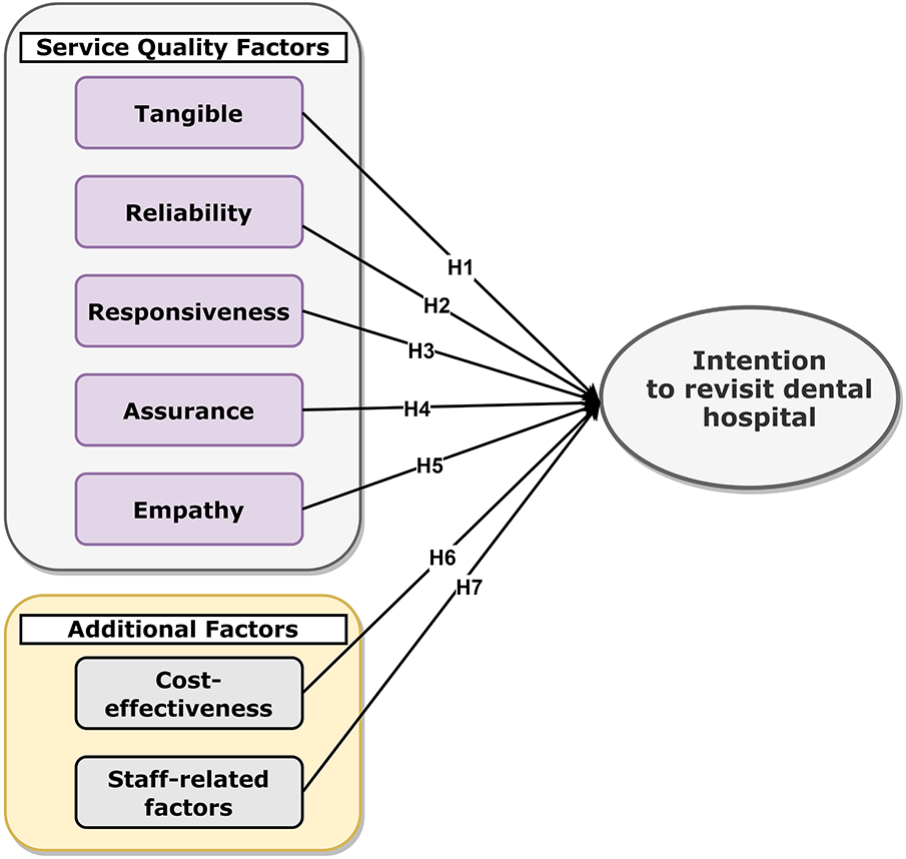
Research model.

The SERVQUAL model was used in this research as a theoretical framework and an instrument for assessing the quality of dental services offered by the dental hospital. The model was chosen due to its widespread recognition in evaluating service quality in hospitals across various sectors (21–24). A study model was developed, as illustrated in Figure 1. The first five factors were derived from the original SERVQUAL model, namely, tangibility, reliability, responsiveness, assurance, and empathy (5). The additional two factors, namely cost-effectiveness and staff-related factors, were added to address the distinctive requirements of the dentistry context. These two factors may have a significant impact on revisit intentions. The revisit intention was the dependent variable (outcome variable) that was included in the model (Figure 1).

### 2.1 Tangibility

Tangibility refers to the visual appeal of physical structures, devices, staff, and communication materials (25). Patients anticipate immaculate and aesthetically pleasing facilities. Although the appearance of clinical settings may not be the foremost determinant of service quality, it does significantly influence how patients perceive the quality of service and satisfaction, particularly when the dental office guarantees outstanding service and a superior experience (26). Also, a previous study found that the appearance of dental staff members in terms of cleanliness and neatness was considered the most important item in the tangibility factor (27).

Therefore, in the context of a dental hospital, the physical attributes of the practice, such as its interior design and atmosphere, and the modern technology and advanced equipment used in the dental hospitals, receptions, and waiting rooms, may have a significant positive effect on the patients' intention to revisit the dental hospital. Based on this context, the following hypothesis is proposed.

H1: Tangibility has a significant positive influence on the patients' intention to revisit the UQU-DTH.

### 2.2 Reliability

Reliability can be defined as the capacity to consistently and precisely provide the committed service (24). Within the dentistry field, this process encompasses all aspects of patients' engagement, including the provision and implementation of necessary services or treatments as promised, as well as the resolution of any issues or concerns that patients have. When patients visit a hospital for dental treatment, they expect the dental practice to be reliable, and the success of a hospital often depends on its ability to satisfy these expectations (28). Prior research has shown inconsistencies in hospitals' capacity to maintain a record free of errors, potentially impacting patients' confidence and contentment in the hospital (20).

Thus, this typically influences the intention of revisiting. This justification leads to the proposition of the following hypothesis:

H2: Reliability has a significant positive influence on the patients' intention to revisit the UQU-DTH.

### 2.3 Responsiveness

This factor demonstrated the commitment and capability of dental hospital staff to provide patients with on-time care. Being a responsive healthcare organization means taking in, evaluating, and quickly addressing requests, comments, queries, and concerns from patients (15). High-quality dental hospital services always reply to patient communications as quickly as possible, which is typically a sign of how much importance the hospital places on patient experience (25).

According to earlier research, responsiveness significantly affects the quality of services (23). Certain dental procedures often require multiple visits. The time between patient assessment and treatment sessions was a key element in the responsiveness factor in the dental settings because of the workload on highly specialized clinics. This might have an effect on the patients' intention to revisit the dental hospital. Therefore, the following hypothesis is formulated.

H3: Responsiveness has a significant positive influence on the patients' intention to revisit the UQU-DTH.

### 2.4 Assurance

Assurance refers to the sense of belief and confidence that patients have in a certain healthcare institution (15). Within the context of dentistry, it is crucial to establish trust in the dental care providers and the organization they're employed for, particularly when it comes to complex dental management that patients may find difficult to comprehend and assess. This trust is essential for ensuring that patients receive the necessary treatment with confidence.

Several studies have shown that the assurance factor was considered the most crucial factor, consistently receiving the highest values when compared to other factors (15, 29). This has a propensity to impact the patients' intention to revisit the dental hospital. Based on this context, the following hypothesis is proposed.

H4: Assurance has a significant positive influence on the patients' intention to revisit the dental hospital.

### 2.5 Empathy

Empathy relates to the manner in which a dental hospital provides its services, creating an atmosphere in the hospital that seems understanding and responsive to the wishes and expectations of its patients (18). Patients' who perceives a hospital's genuine concern for their welfare is inclined to exhibit more loyalty and revisit intention towards that dental hospital (16). Furthermore, it elucidates the way in which the organization offers care and individualized attention to its patients. Therefore, the following hypothesis is formulated.

H5: Empathy has a significant positive influence on the patients' intention to revisit the UQU-DTH.

### 2.6 Cost-effectiveness

This factor pertains to the patients' perception that the dental services provided by the dental hospital are reasonably priced and offer satisfactory value in relation to the amount of money spent (29). Prior research has shown that the cost of dental treatments was the most significant factor influencing the success of a dental business. Additionally, it was considered a factor that contributed to patient satisfaction (30). The affordability of dental care services plays a crucial role in shaping consumers' choices to use dental clinics and it is essential that the cost of dental care remains reasonable and accessible (23). Also, the availability of affordable healthcare services for patients may serve as a gauge of service quality (14).

Therefore, it may be inferred that if the patients are satisfied with the costs, they are more inclined to revisit and endorse the service to others. Based on this context, the following hypothesis is proposed.

H6: Cost-effectiveness has a significant positive influence on the patients' intention to revisit the UQU-DTH.

### 2.7 Staff-related factors

This factor pertains to the personality traits and professionalism of the staff, including their expertise, professional abilities, and ability to explain treatment plans clearly to patients. According to a previous study, patients' perceptions of the dentists' skills are crucial, and some patients show low confidence in dental students (30). Also, a previous study showed that patients consider professional expertise and personal characteristics, such as gender, age, and ethnicity, as influential factors when selecting a dentist (31).

Furthermore, another study explained that among the primary determinants that determine patients' attitudes towards dentists are the ethical conduct of the dentist, an accurate diagnosis and high-quality treatment, and the communication proficiency of the dentist (32). Thus, the following hypothesis is formulated.

H7: Staff-related factors has a significant positive influence on the patients' intention to revisit the UQU-DTH.

### 2.8 Revisit intention

This factor can be described as the extent to which hospital administrators have formulated strategies, such as delivering high-quality services and creating a positive experience, to encourage specific behaviors in patients with the aim of fostering long-term relationships (14–16). This, in turn, leads to patients returning to the same healthcare provider for their health care requirement. Given the significance of patients' revisit intentions for hospital management in terms of competitiveness, credibility, and revenue (14). In the dental context, revisit intention can be referred to a patient's desire to return to the same dental care facility to receive dental treatment.

The concept of revisit intention encompasses two primary items: the desire or readiness to recommend the hospital to others, and the intention or readiness to return to the hospital. This research has chosen revisit intention as the dependent variable. This leads us to propose the following hypothesis.

H8: Overall, the proposed extended SERVQUAL model significantly predicts patients' behavioral intention to revisit the UQU-DTH.

## 3 Materials and methods

### 3.1 Ethical consideration

Authorization to carry out this study was obtained from the Research and Ethics Committee of Umm Al-Qura University Approval No. (HAPO-02-K-012-2023-05-1635).

### 3.2 Study setting and subjects

The cross-sectional study was conducted from September to November 2023 in the outpatient waiting areas and clinical settings of the Umm Al-Qura University Dental Teaching Hospital at (UQU-DTH). The research participants were drawn from the patient population who sought dental care at the hospital. The research included patients who were over 18 years old and accepted the request to participate, as well as those who visited the hospital once or multiple times. The research excluded patients who lacked the ability to read or comprehend the questionnaire and those who were below the age of 18. The method of convenience sampling was used to provide a proximately representative proportion of patients to ensure that the recruited sample will be represented as much as possible. To determine the minimum sample size for the study, the Raosoft online sample calculator was used. With a margin of error of 5%, a confidence interval of 95%, a response distribution of 50%, and a population of approximately 2,000, the recommended sample size was 323 patients. In order to account for missing data or incomplete responses, the sample size was 10% inflated, and 355 patients were invited to participate in the study.

### 3.3 Research instrument

The data for this research was collected using an Arabic version of the SERVQUAL instrument (20). The Arabic questionnaire was used due to the predominant number of patients who are native Arabic speakers. The items related to the additional factors, specifically cost-effectiveness, staff-related factors, and revisit intention, were derived from prior empirical studies (14–16). A committee consisting of four faculty members and educators from the authors' dental school was invited to check the validity of the final version of the questionnaire and thoroughly examine its wording, semantics, phrasing, and experiential aspects. Then, the questionnaire was piloted with a group of 20 patients who were not part of the research. Following the pilot inquiry, some words were modified.

### 3.4 Data collection methods

An online platform (http://forms.microsoft.com) was used to distribute the questionnaire to patients who attended the hospital during business hours. The questionnaire contains the following sections: The first section contains a participant information sheet. The second section included consent statements. The third section contains demographic questions, including gender, age, nationality, education level, type of visit, place of residence, and reason for visiting. The fourth section included 32 service-quality items. The responses measured using a 5-point Likert scale (1 = strongly disagree to 5 = strongly agree).

### 3.5 Data management and analysis plan

The distribution of the questionnaire scores was determined using descriptive statistics and frequency analyses (mean and standard deviation). The reliability (internal consistency) of the questionnaire items in each factor was examined using Cronbach's alpha (33). The study used hierarchical regression analysis to investigate the relationship between the independent factors and the dependent factor (revisit intention) while accounting for the impact of demographic variables (34). Variance inflation (VIF) analysis was used to measure multicollinearity; results of less than 10 indicate the absence of multicollinearity. The results imply that there are no problems with multicollinearity. The analysis was conducted using IBM, Inc.'s SPSS Version 29.0.

## 4 Results

### 4.1 Demographic characteristics of participants

A total of 330 completed responses were received, with a 93% response rate. The participants were comprised of 61.5% males and 38.5% females. Of these, 63.3% were under the age of 35, and 36.7% were over the age of 35. Forty percent of the participants had a university education, while 24.2% and 35.8% were in primary/secondary and high school level groups, respectively. Of the 330 patients, 59.1% came for multiple visits, while 40.9 came for the first and second time. In addition, 83.9 percent of participants came for dental treatment, while 10.9% and 5.2% came for check-up/consultation and emergency care, respectively. The majority of participants 72.1% were treated by undergraduate dental students. The remaining participants received dental care from dental interns, postgraduate dentists, and faculty members, with 17%, 4.8%, and 6.1%, respectively. Furthermore, 58.5% were non-Saudis, while 41.5 were Saudis.

### 4.2 Reliability analysis

Cronbach's alpha scores shown in Table 1 indicated that each factor exhibited strong internal reliability. All factors showed excellent alpha (*α*) scores with a range from 0.796 to 0.922.

**Table 1:** Descriptive statistics for model factors and its measuring scale items.

Factor	Measurement variables	Mean (SD)	Cronbach's alpha (*α*)
Tangible	The dental hospital has up to date dental equipment.	4.41 (.843)	.863
	The dental hospital has visual appeal and comfortable physical facilities.	4.27 (.968)	
	Staff show neat professional appearance.	4.61 (.793)	
	The dental hospital has clean facilities.	4.58 (.733)	
	**Average Tangible scores**	4.46 (.705)	
Reliability	The dental hospital provide service as promised.	4.52 (.753)	.866
	Staff show sincere interest in solving patients’ problems.	4.64 (.694)	
	The dental hospital perform the service right the first time.	4.57 (.726)	
	The dental hospital provide services at the time promised.	4.39 (.904)	
	The dental hospital maintains error-free records.	4.47 (.822)	
	**Average Reliability scores**	4.51 (.632)	
Responsiveness	The dental hospital inform patients when services will be performed.	4.40 (.817)	.839
	Staff give prompt service to patients.	4.21 (.940)	
	Staff willingness to help patients.	4.60 (.704)	
	Staff readily respond to patients’ request.	4.52 (.837)	
	**Average Responsiveness scores**	4.43 (.680)	
Assurance	Patients feel safe during their treatment.	4.51 (.800)	.888
	Patients feel Trustworthy of dental staff.	4.50 (.757)	
	Staff are courteous at all times.	4.65 (.659)	
	The dental hospital pays attention to training of staff's professional knowledge and skills.	4.61 (.663)	
	**Average Assurance scores**	4.56 (.625)	
Empathy	The dental hospital show attention to individual patient.	4.61 (.708)	.916
	The dental hospital give personalized care to patient.	4.63 (.660)	
	Staff recognize of specific needs of patients.	4.63 (.658)	
	The dental hospital has convenient operating hours.	4.44 (.921)	
	Staff treat patient with dignity and respect.	4.72 (.580)	
	**Average Empathy scores**	4.60 (.618)	
Cost effectiveness	The dental provides quality dental services at a reasonable price.	4.47 (.949)	.796
	The dental hospital provides dental services free of charge.	4.70 (.636)	
	At the current price, hospital provides a high quality dental care in a good value.	4.52 (.822)	
	**Average cost effectiveness scores**	4.56 (.684)	
Staff-related factors	Staff are professional and knowledgeable.	4.55 (.710)	.922
	The dentist clearly explained my treatment plan.	4.61 (.733)	
	The dentist answered my questions.	4.64 (.707)	
	Staff has sufficient clinical skills.	4.65 (.679)	
	**Average staff related factors scores**	4.61 (.636)	
Revisit intention	I intend to continue visiting this dental hospital for dental services.	4.69 (.580)	.878
	I will recommend the hospital to relatives and friends.	4.58 (.700)	
	I consider this dental hospital to be my first choice in case I need dental treatment again.	4.63 (.646)	
	**Average revisit intention**	4.63 (.652)	

### 4.3 The perception of the participants about the extended SERVQUAL model factors

The descriptive statistics for each factor are shown in Table 1. All measurements were on a 5-point Likert scale. The means of the responsiveness and tangible factors were ranked as the lowest, with means scores of 4.43 and 4.46, respectively. The mean score of revisit intention was the highest, at 4.63. Also, “*staff give prompt service to patients*” and “*the hospital has visual appeal and comfortable physical facilities*” items had the lowest mean scores at 4.21 and 4.27 respectively. The item “*Staff treat patient with dignity and respect*” had the highest mean scores, at 4.72 (Table 1).

### 4.4 Hierarchical regression analysis

#### 4.4.1 Testing of assumptions

In order to verify that the assumptions of multiple regression analysis were satisfied, initial evaluations were performed. Initially, scatter plots connecting the independent variables to the dependent variable demonstrated that all relationships met the linearity requirement. Using the Durbin-Watson statistic, the independence of error terms was examined; the result was 1.993, which is close to the critical value of 2.0 (34). The absence of multicollinearity assumption was also tested and satisfied using the variance inflation factor index, which was within the recommended threshold values of 1 and 10 (33, 34). The standardized residuals histogram and normal P–P plot were employed to ascertain normality. All of the assumptions were met.

#### 4.4.2 Control variables

Demographic variables, i.e., gender, age, level of education, and nationality, were included as control variables. Also, other status variables such as type of visit, reason for visit, and type of dentist were controlled and included in this study. A total of seven variables were therefore considered control variables.

#### 4.4.3 Results of the hierarchical linear regression analysis

As shown in Table 2, the control variables were first entered as Model 1 (revisit intention = gender + age + education level + nationality + type of visit + reason for visit + type of dentist) of the hierarchical regression equation. Findings indicated a significant model (*F* = 3.468, *p* < 0.001) and showed that these variables account for 7% (*R*^2^ = 0.07) of the variation in revisit intention to dental teaching hospital.

**Table 2:** Hierarchical regression analysis (model summary).

Model	*R*	*R* square	Adjusted *R* square	Std. error of the estimate	*R* square change	*F* change	df1	df2	Sig. *F* change
1	.265	.070	.050	.635	.070	3.468	7	322	.001*
2	.810	.656	.641	.390	.586	76.608	7	317	.001*

* *p*-value < 0.05.

Subsequently, Model 2 was utilized to assess the impact of the seven extended SERVQUAL model factors—tangibles, reliability, responsiveness, assurance, empathy, cost-effectiveness and staff-related factors —on the prediction of intention to revisit the dental teaching hospital. The inclusion of these variables yielded a statistically significant *R*^2^ change of 58.6% (*ΔR*^2^ = 0.586) and an overall significant model [*F* (14, 315) = 42.888, *p* < 0.001] (*ΔF* = 76.608, *p* < 0.001) (Table 2).

The preliminary and *t*-statistic values in Model 1 demonstrated that male and female respondents did not differ in their revisit intention (*β* = 0.25, *t* = 0.445, *p* = 0.656). Age and level of education variables, on the other hand, have a significant impact on participants' revisit intentions. Respondents over the age of 35 have a higher level of revisit intention to dental teaching hospital than those under the age of 35 (*β* = .13, *t* = 2.842, *p* = 0.005). In addition, participants with a university degree were less likely to revisit dental hospital than those with a school degree (*β* = −0.142, *t* = −2.473, *p* = 0.014) (Table 3).

**Table 3:** Hierarchical regression analysis (coefficients).

Predictor variables	Unstandardized coefficients		Standardized coefficients	*t*	*p*-value	Collinearity statistics	
	*B*	*SE*	*β*			Tolerance	VIF
Model 1							
(Constant)	4.544	.111		41.100	<.001*		
Gender	.034	.076	0.25	.445	.656	.906	1.103
Age	.214	.075	.158	2.842	.005*	.934	1.071
Education level	−.189	.077	−.142	−2.473	.014*	.870	1.150
Nationality	.135	.076	.102	1.766	.078	.864	1.158
Type of visit	.087	.074	.066	1.185	.237	.935	1.069
Reason of visit	−.104	.099	−.059	−1.048	.295	.921	1.086
Type of dentist	−7.729	.118	.000	−.001	.999	.905	1.105
Model 2							
(Constant)	.426	.195		2.181	.030		
Gender	.139	.047	.104	2.972	.003*	.026	.165
Age	.003	.049	.002	.064	.949	.165	.004
Education level	−.043	.048	−.033	−.900	.369	−.151	−.051
Nationality	.015	.048	.011	.310	.757	.157	.017
Type of visit	.080	.046	.060	1.743	.082	.073	.098
Reason of visit	−.119	.063	−.067	−1.897	.059	−.017	−.106
Type of dentist	−.047	.074	−.023	−.639	.523	−.030	−.036
Tangibles	.039	.052	.042	.759	.448	.611	.043
Reliability	.112	.075	.109	1.502	.134	.718	.084
Responsiveness	.174	.057	.181	3.027	.003*	.696	.168
Assurance	.090	.068	.087	1.339	.182	.691	.075
Empathy	.076	.076	.072	1.003	.317	.713	.056
Cost effectiveness	.156	.043	.164	3.588	<.001*	.614	.198
Staff related factor	.274	.071	.268	3.867	<.001*	.740	.213

* *p*-value < 0.05.

The final *β* and *t*-statistic values in Model 2 indicated that the demographic variables were no longer significant when the theoretical factors of the service quality model were added to the model except for gender. The responsiveness factor had a significant and positive effect on revisit intention (*β* = 0.181, *t* = 3.027, *p* = 0.003), confirming hypothesis H3. Furthermore, it was found that both cost-effectiveness (*β* = 0.164, *t* = 3.588, *p* < 0.001) and staff-related factors (*β* = 0.268, *t* = 3.867, *p* < 0.001) have a statistically significant positive impact on revisit intention. These findings lend support to hypotheses H6 and H7 (Table 3).

Also, results testing showed that the overall extended SERVQUAL model could predict the revisit intention *F*(4, 315) = 42.888, *R*^2^ = 65.6, *p* < 0.001) and supported hypothesis H8. The service quality factors were shown to explain 65.6% of the variance in revisit intention. The standardized regression coefficients indicated that the strongest determinant of revisit intention was staff related factors, followed by cost effectiveness and responsiveness. Finally, no significant values were identified in the model, so hypotheses H1, H2, H4, and H5 were rejected (Table 3).

## 5 Discussion

The aim of this empirical study was to examine the influence of the extended SERVQUAL model on the intention of revisiting a dental teaching hospital. The study addressed the research question by determining the salient factors affecting revisit intention among adult patients.

This study found that the staff-related factors dimension was the most salient predictor of patients' intentions to revisit. More precisely, this factor explained 26.8% of the variation in revisit intention. The finding was interesting, indicating that patients' perceptions of dentists' knowledge, clinical expertise, and interpersonal skills were significant factors influencing their revisit intention. One potential reason for this might be because the majority of patients included in this study received treatment from dental students and interns. The dental students are required to allocate a substantial amount of time towards the processes of diagnosis, treatment planning, consultations, and executing treatments while being closely monitored by clinical instructors (35). Patients may have a preference for and feel assured in this setting (36). This finding aligns with a prior study carried out in a dental teaching hospital in Hong Kong, which found that patients regarded the following factors as important indicators of quality dental care: the extent of information provided to patients regarding the disease, treatment procedure, and potential complications, and the use of comforting and encouraging language during treatment (30). Similarly, a study conducted at a dental hospital in the USA found that patients expressed satisfaction with the level of dental care delivered by dental students (9). They reported that the student providers demonstrated expertise in addressing their treatment requirements and effectively explained the treatment procedure in a courteous way prior to commencing the treatment (9). In other words, when students and clinical instructors thoroughly analyze a patient's case, considering all treatment aspects and options, it provides a clear and complete treatment plan that addresses all of the patient's concerns, which will positively influence their intention to revisit the center.

The results of this research indicated that the responsiveness factor has a substantial and positive impact on patients’ intentions to revisit. Specifically, this factor accounts for 18.1% of the variation in revisit intention. This highlighted the crucial need for dental hospitals to promptly address patients' inquiries and apprehensions, particularly in contemporary high-speed society. In addition, patients' expectations that the dental inquires will be completed punctually and efficiently are crucial to revisit intention. This outcome was contrary to several previous studies that found a responsiveness factor had a positive influence on revisit intention but was not significant (16, 24, 27). A prior study revealed that dental patients in Indonesia didn't perceive responsiveness as a significant determinant of dental clinic service quality (27). Nevertheless, a prior study carried out in Saudi Arabia revealed that the responsiveness component had a substantial influence on patients' satisfaction with hospital services (17). Future research endeavors may take into account the impact of cultural variations on patients' perspectives.

Another crucial finding is that the cost-effectiveness factor has a significant positive influence on patients revisit intention. In particular, this factor explains 16.4% of the variance in revisit intention. The plausible explanation is that the dental teaching hospital offered a significant portion of dental care for free or at a low cost, particularly for complex dental procedures such as dental implants. This result was in line with a previous study conducted in Malysia that found that implementing pricing strategies may effectively influence patients' behavioral intentions in a positive manner (14). A significant proportion of the population in several countries is unable to afford dental healthcare as a result of the escalating expenses associated with dental materials and operations, compelling dentists to increase their rates (37, 38). Due to the exorbitant expenses associated with dental treatment, patients might seek treatment from public hospitals (39). As a result, the cost-effectiveness factor was a crucial factor in patients' revisit intention.

The mean scores for each extended SERVQUAL factor were high in this sample, indicating that patients expressed contentment with the quality of service provided at the dental hospital. Furthermore, group means of empathy and staff-related factors were the highest and rated as the most agreed factors. This finding aligns with prior research undertaken in Saudi Arabia, which showed that patients regarded empathy as a satisfactory criterion (20).

However, tangibles and responsiveness were rated as the least agreed. This suggested that patients had expressed concerns about the specific service the hospital offered, particularly regarding their flexibility, openness, and promptness. The possible justification for this might be the difficulty of delivering dental care within the required timeframe for the patient due to the need to follow a series of processes, including the opening of files, initial assessment and meticulous treatment plans, and triage, before reaching the dental students for treatment. This finding was consistent with an earlier study that revealed dental patients expressed dissatisfaction with the duration of waiting time at the dental clinic, highlighting a significant issue that requires resolution (40). A possible solution may include but not limited to enhance the capacity of dental chairs, extend working hours, and decrease the number of patients, or increase number of clinical instructors in order to shorten wait times. However, it is challenging to implement such solutions at such public teaching hospital because of the large number of patients in need of dental care and the facility's fixed working hours. Financial concerns would be another obstacle to implement such solutions.

Regarding the tangible factor, dental patients may have been concerned about the available infrastructure at the dental hospital, including dental equipments, aesthetic appeal, and pleasant physical amenities. This finding was consistent with prior research carried out at public hospitals in Saudi Arabia (17, 20). A research conducted in the Eastern Province of Saudi Arabia revealed that patients experienced the most significant gap in quality when it came to tangibles (20). Similarly, another study carried out in the southern area of Saudi Arabia revealed that patient satisfaction is influenced by factors such as the quality of physical facilities, equipment, and the appearance of physicians and staff members (17). It is pivotal to emphasize that the dental hospital in this study is government-owned for public services and training dental students, and not driven by commercial motives. However, the average Tangible scores were not at the level that would raise a significant concern at the dental center.

### 5.1 Study limitations

The limitations of this research center on its geographical viability, since it was restricted to a single public dental teaching hospital. As such, it may not accurately reflect the opinions of Saudi patients who have attended privately held dental hospitals. A comprehensive picture might be obtained from future research comparing the quality of care offered by public and private hospitals. Furthermore, it is important to note that the patient's revisit intention may vary as time progresses. Therefore, a cross-sectional design may not be appropriate for effectively monitoring and analyzing these changes. A future longitudinal study may provide a better grasp of the factors investigated. Additionally, using a convenience sample strategy may restrict our capacity to generalize findings to a broader population.

### 5.2 Study implications and recommendations

This study showed that the added factors “cost-effectiveness” and “staff-related factors” were useful in predicting patients' revisit intentions. They should be added to the SERVQUAL model when it is used for assessing dental services. The proposed model has never been used in the dental services context and could be utilized to provide a solid theoretical foundation for service quality and employed as a robust theoretical framework for assessing service quality. At the level of the organization, the approach may be used to constantly assess and regulate the quality of services. Additionally, it may be used for the aim of developing hospital services, including the formulation of initial objectives and targets, the execution of operational strategic administration, and future-oriented competitive advertising. Moreover, it assists in identifying the specific areas that need standardization and improvement.

The primary recommendations of this research indicate that staff-related factors have a significant impact on the probability of patients revisiting the hospital. Thus, it is essential for dental institutions to prioritize the recruitment of staff that possess exceptional expertise, vast knowledge, and excellent interpersonal skills for effective patient communication. In dental clinics, since the duration of dental treatment is often longer than in regular medical treatments, effective communication between dentists and patients is crucial. Another crucial recommendation is the need to increase the number of public dental hospitals. The government in Saudi Arabia provides coverage for the majority of dental care at the public dental hospital. Nevertheless, the overwhelming demand placed on the public hospital results in significant delays in accessing dental care. Finally, Moreover, including these criteria in the assessment models, auditing strategies, procedures, and policies of dental hospitals in a uniform manner is expected to enhance patient care processes and improve the overall quality performance of hospitals.

## 6 Conclusion

In conclusion, this study's analysis determined that the primary service quality factors that have a substantial impact on patients' revisit intention include responsiveness, cost effectiveness, and staff-related factors. In the current research, all the service quality variables were found to be very close to meeting the patients' agreement, indicating the effectiveness of the dental hospital's efforts in meeting the service quality standards. However, focus should be given to improving physical facilities and equipment, as well as addressing responsiveness issues, in order to further enhance the delivery of high-quality dental services.

## Data Availability

The original contributions presented in the study are included in the article/Supplementary Material, further inquiries can be directed to the corresponding author.

## References

[1] Righolt AJ Sidorenkov G Faggion CM Jr Listl S Duijster D. Quality measures for dental care: a systematic review. Community Dent Oral Epidemiol. (2019) 47:12–23. 10.1111/cdoe.12429 30375669 PMC7379624

[2] Lin Y Hong YA Henson BS Stevenson RD Hong S Lyu T Assessing patient experience and healthcare quality of dental care using patient online reviews in the United States: mixed methods study. J Med Internet Res. (2020) 22:e18652. 10.2196/18652 32673240 PMC7380989

[3] Gray L McNeill L Yi W Zvonereva A Brunton P Mei L. The “business” of dentistry: consumers’ (patients’) criteria in the selection and evaluation of dental services. PLoS One. (2021) 16:e0253517. 10.1371/journal.pone.0253517 34358252 PMC8345823

[4] Mohammad MA. Healthcare service quality: towards a broad definition. Int J Health Care Qual Assur. (2013) 26:203–19. 10.1108/09526861311311409 23729125

[5] Parasuraman A Zeithaml VA Berry LL. A conceptual model of service quality and its implications for future research. J Mark. (1985) 49:41–50. 10.1177/002224298504900403

[6] Aldossary MS. Dental governance and the Saudi vision 2030: a narrative review. Saudi J Health Syst Res. (2022) 5:1–7. 10.1159/000526361

[7] Adebayo E Adesina B Ahaji L Hussein N. Patient assessment of the quality of dental care services in a Nigerian hospital. J Hosp Adm. (2014) 16:3. 10.5430/jha.v3n6p20

[8] Al-Badawi A Al-Qahtani E. Use servqual’s perception and expectation model to measure the quality of educational services in public education schools in urban Abha. Al-Azhar J Educ. (2019) 38:11–49. 10.21608/jsrep.2019.78731

[9] Rai NK Tyrrell H Carey C Tiwari T. Patient perceptions in quality of care: report from university veterans clinic. BMC Oral Health. (2019) 19:268–77. 10.1186/s12903-019-0971-6 31796009 PMC6892177

[10] Tashkandi F Hejazi L Lingawi H. Patients’ satisfaction with dental care services provided by educational dental hospital. Int J Health Sci Res. (2017) 7:135–42. Available online at: https://www.ijhsr.org/IJHSR_Vol.7_Issue.6_June2017/21.pdf (Accessed December 15, 2023).

[11] Health Sector Transformation Program. In Vision 2030. (2023). Available online at: https://www.vision2030.gov.sa/en/vision-2030/vrp/health-sector-transformation-program/ (Accessed December 15, 2023).

[12] Park SJ Yi Y Lee YR. Multiplicative versus additive models in measuring service quality. Total Qual Manag Bus Excell. (2023) 34:2105–23. 10.1080/14783363.2023.2229272

[13] Akdere M Top M Tekingündüz S. Examining patient perceptions of service quality in Turkish hospitals: the SERVPERF model. Total Qual Manag Bus Excell. (2020) 31:342–52. 10.1080/14783363.2018.1427501

[14] Lai KP Yuen YY Chong SC. The effects of service quality and perceived price on revisit intention of patients: the Malaysian context. Int J Qual Serv Sci. (2020) 12:541–58. 10.1108/IJQSS-02-2019-0013

[15] Lee S Kim EK. The effects of Korean medical service quality and satisfaction on revisit intention of the United Arab Emirates government sponsored patients. Asian Nurs Res. (2017) 11:142–9. 10.1016/j.anr.2017.05.008 28688500

[16] Wandebori H. Revisit intention to hospital: factors unveiled from a case study of Balimed hospital. J Manaj Teori Dan Terap J Theory Appl Manag. (2017) 10:205–16. 10.20473/jmtt.v10i3.3551

[17] Alghamdi FS. The impact of service quality perception on patient satisfaction in government hospitals in Southern Saudi Arabia. Saudi Med J. (2014) 35:1271–3. 25316476 PMC4362118

[18] Alanazi E Alanazi H Alanazi M Alsadoun A Asiri S Bahari G. Quality perceptions, expectations, and individual characteristics among adult patients visiting primary healthcare centers in Saudi Arabia: a cross-sectional study. Healthcare. (2023) 11:208–16. 10.3390/healthcare11020208 36673576 PMC9859356

[19] Al-Borie HM Sheikh DAM. Patients’ satisfaction of service quality in Saudi hospitals: a SERVQUAL analysis. Int J Health Care Qual Assur. (2013) 26:20–30. 10.1108/09526861311288613 23534103

[20] Fraihi KJA Famco D Famco F Latif SA. Evaluation of outpatient service quality in Eastern Saudi Arabia. Saudi Med J. (2016) 37:420–8. 10.15537/smj.2016.4.14835 27052285 PMC4852020

[21] Sitaraman P Shanmugasundaram K Muthukrishnan A. Assessment of service quality in special care dentistry department using SERVQUAL model. J Indian Acad Oral Med Radiol. (2020) 32:209–14. 10.4103/jiaomr.jiaomr_69_20

[22] Li M Lowrie DB Huang CY Lu XC Zhu YC Wu XH Evaluating patients’ perception of service quality at hospitals in nine Chinese cities by use of the ServQual scale. Asian Pac J Trop Biomed. (2015) 5:497–504. 10.1016/j.apjtb.2015.02.003

[23] Bahadori M Raadabadi M Ravangard R Baldacchino D. Factors affecting dental service quality. Int J Health Care Qual Assur. (2015) 28:678–89. 10.1108/IJHCQA-12-2014-0112 26241090

[24] Rocha J Pinto A Batista M Paula Jd Ambrosano G. The importance of the evaluation of expectations and perceptions to improve the dental service quality. Int J Health Care Qual Assur. (2017) 30:568–76. 10.1108/IJHCQA-01-2016-0008 28714836

[25] Ramez DWS. Patients’ perception of health care quality, satisfaction and behavioral intention: an empirical study in Bahrain. IJBSS. (2012) 3:18. Available online at: https://ijbssnet.com/journals/Vol_3_No_18_Special_Issue_September_2012/15.pdf (Accessed December 15, 2023).

[26] Ali DA. Patient satisfaction in dental healthcare centers. Eur J Dent. (2016) 10:309–14. 10.4103/1305-7456.184147 27403045 PMC4926580

[27] Akbar FH Pasinringi S Awang AH. Factors affecting dental center service quality in Indonesia. Pesqui Bras Em Odontopediatria E Clínica Integrada. (2019) 10:e4269. 10.4034/PBOCI.2019.191.53

[28] Mirandani. Dental care service quality assists in comprehensive clinical dental risk management: A narrative review.. Available online at: https://www.jioh.org/article.asp?issn=0976-7428;year=2022;volume=14;issue=3;spage=209;epage=214;aulast=Mirandani (Cited December 12, 2023).

[29] Siripipatthanakul S Bhandar M. A qualitative research factors affecting patient satisfaction and loyalty: a case study of smile family dental clinic. Int J Trend Res Dev. (2021) 5:877–96. Available online at: https://www.ijtsrd.com/papers/ijtsrd44975.pdf (Accessed December 15, 2023).

[30] Luo JYN Liu PP Wong MCM. Patients’ satisfaction with dental care: a qualitative study to develop a satisfaction instrument. BMC Oral Health. (2018) 18:15. 10.1186/s12903-018-0477-7 29382318 PMC5791245

[31] Furnham A Swami V. Patient preferences for dentists. Psychol Health Med. (2009) 14:143–9. 10.1080/13548500802282690 19235073

[32] Belén R Andrés R. Patients’ perceptions about dentists a literature review. Odontoestomatología. (2016) 18:15–22. Available online at: http://www.scielo.edu.uy/pdf/ode/v18n27/en_v18n27a03.pdf (Accessed December 16, 2023).

[33] Sharka R San Diego J Nasseripour M Banerjee A. Factor analysis of risk perceptions of using digital and social media in dental education and profession. J Dent Educ. (2023) 87:118–29. 10.1002/jdd.13085 36036230

[34] Field A. Discovering Statistics Using IBM SPSS Statistics. Thousand Oaks, CA: SAGE Publications Ltd (2017). p. 32.

[35] Motloba PD Ncube O Makwakwa LN Machete ML. Patient waiting time and satisfaction at a tertiary dental school. South Afr Dent J. (2018) 73:400–5. 10.17159/2519-0105/2018/v73no6a3

[36] Sharka R San Diego JP Nasseripour M Banerjee A. Identifying risk factors affecting the usage of digital and social media: a preliminary qualitative study in the dental profession and dental education. Dent J. (2021) 5:53. 10.3390/dj9050053 PMC8151279 34066871

[37] Sharka R Abed H Hector M. Oral health-related quality of life and satisfaction of edentulous patients using conventional complete dentures and implant-retained overdentures: an umbrella systematic review. Gerodontology. (2019) 36:195–204. 10.1111/ger.12399 30875108

[38] Sharka R. Psychometric properties of the Arabic version of the perceived prosthodontic treatment need scale: exploratory and confirmatory factor analyses. PLOS One. (2024) 19:e0298145. 10.1371/journal.pone.0298145 38319938 PMC10846707

[39] Bouchard P Renouard F Bourgeois D Fromentin O Jeanneret Mh Beresniak A. Cost-effectiveness modeling of dental implant vs. Bridge. Clin Oral Implants Res. (2009) 20:583–7. 10.1111/j.1600-0501.2008.01702.x 19530315

[40] Dewi FD Sudjana G Oesman YM. Patient satisfaction analysis on service quality of dental health care based on empathy and responsiveness. Dent Res J. (2011) 8:172–7. 10.4103/1735-3327.86032 PMC3221083 22135687

